# Unveiling the bacterial community structure and soil properties in bulk and rhizospheric compartments of *Tamarix gallica* from an arid Algerian saline ecosystem

**DOI:** 10.3389/fmicb.2026.1731684

**Published:** 2026-08-10

**Authors:** Hanaa Abdelbari, Natalia Rodríguez-Berbel, Raúl Ortega, Isabel Miralles, Yassine Moustafa Mahdad, Semir Bechir Suheil Gaouar

**Affiliations:** 1 Applied Genetics in Agriculture, Ecology and Public Health Laboratory, University of Tlemcen, Tlemcen, Algeria; 2 Department of Ecology and Ecosystem Management, TUM School of Life Sciences Weihenstephan, Chair of Soil Science, Technical University of Munich, Freising, Germany; 3 Research Unit Comparative Microbiome Analyses, Helmholtz Zentrum München, Munich, Germany; 4 Department of Agronomy & Center for Intensive Mediterranean Agrosystems and Agri-Food Biotechnology (CIAIMBITAL), University of Almeria, Almería, Spain; 5 Laboratory for Sustainable Management of Natural Resources in Arid and Semi-Arid Areas, University Center of Naâma, Naâma, Algeria

**Keywords:** 16S rRNA amplicon study, halophyte-associated microbiomes, plant soil interactions, soil properties, soil salinization

## Abstract

Soil salinization is among the most critical threats to agriculture and food security, particularly in arid and semi-arid regions. Despite their ecological and agricultural significance, the microbial processes in saline soils of arid regions remain poorly characterized. This first 16S rRNA amplicon-based study of Algerian saline soils examines bacterial communities and physico-chemical properties in the Naama salt flat, focusing on both bulk and rhizosphere soils associated with *Tamarix gallica* L. Bacterial diversity and community composition were analyzed through high-throughput 16S rRNA gene sequencing on an Illumina MiSeq platform, and soil parameters, including salinity, nutrients, organic carbon, and water retention were measured. Sequence data were processed with QIIME2, and multivariate analyses were applied to compare soil types and explore correlations between taxa and soil properties. Rhizosphere soils tended towards higher Na^+^, K^+^, and Ca^2+^ levels, whereas bulk soils showed greater phosphorus availability. Bacterial diversity was lower in the rhizosphere, which was characterized by a distinct taxonomic composition, including taxa such as *Promicromonospora* and *Nocardioides* potentially involved in nutrient cycling and stress adaptation. Correlation analyses revealed microbial adaptations to salinity and nutrient availability. This study lays the groundwork for developing plant growth-promoting rhizobacteria aiming to combat land degradation and contribute to long-term food security and environmental resilience in arid regions.

## Introduction

Soil salinization is a global environmental challenge, affecting over 1.5 billion hectares of land, with irrigated agricultural areas experiencing significant productivity losses of approximately $27 billion annually (Shahid et al., 2018). Soil salinization reduces crop yields, depletes freshwater resources, disrupts soil microbial communities, and leads to the loss of biodiversity in ecosystems, making it one of the major threats to global food security and environmental sustainability (Sanga et al., 2024). The main drivers of salinization include unsustainable irrigation practices, inadequate drainage, rising sea levels, and excessive evaporation rates in arid and semi-arid climates (Wicke et al., 2011). In the Mediterranean region, particularly in semi-arid areas with intensive agriculture, salinization is a widespread and growing issue driven by irrigation mismanagement, high evaporation, and insufficient drainage (Qadir et al., 2014; Shahid et al., 2018; Jacobs et al., 2019). In Algeria, salinity poses major challenges in arid and semi-arid zones, particularly within endorheic depressions, where soils exhibit hypersalinity, nutrient imbalances, and poor fertility (Laoufi et al., 2023).

However, there are habitats like salt flats, which are hypersaline ecosystems that host halophyte vegetation and specialized microbial communities adapted to extreme osmotic stress (Dorador et al., 2013). Among these halophytes, *Tamarix gallica* L. is notable for its salinity tolerance and ability to thrive in poor soil conditions (Terrones et al., 2016). This species is naturally distributed across the Mediterranean basin and North Africa, colonizing coastal marshes, riverbanks, and inland salt flats where few other plants survive (Zahran and Willis, 2009). Recent studies highlight its ecological and symbiotic potential: inoculation with native arbuscular mycorrhizal fungi significantly enhanced secondary metabolite content and stress tolerance in *T. gallica* grown in saline soils (Bencherif et al., 2024), while bacteria isolated from its rhizosphere, such as *Pseudomonas* sp. TR47, have demonstrated strong plant growth-promoting effects under salt stress (Bakelli et al., 2025). Its deep root system enhances soil stability, while its ability to excrete excess salts contributes to salinity mitigation and overall ecosystem resilience (Zahran and Willis, 2009) which makes it a valuable model species for exploring plant–microbe interactions in extreme environments. The microbial communities thrive in extreme saline environments by regulating osmotic balance, producing stress-mitigating compounds, and enhancing nutrient availability through biological processes such as nitrogen fixation and phosphorus solubilization (Morcillo and Manzanera, 2021). Other studies have shown that salt-tolerant microbes can significantly improve soil fertility and plant growth in saline soils by modifying rhizospheric conditions and supporting plant-microbe interactions (AbuQamar et al., 2024). These interactions can serve as a foundation for sustainable strategies to rehabilitate degraded soils, emphasizing the importance of focused investigations into Algerian salt flat microbiomes ecosystems that remains underexplored.

Microbial activity and nutrient availability are strongly influenced by soil physico-chemical properties such as salinity, acidity, and water retention (Zhalnina et al., 2018). Salinity can inhibit microbial growth by creating osmotic stress, reducing microbial biomass, and altering community composition (Yan et al., 2015). Acidity affects microbial communities by changing species composition and decreasing nutrient availability, as certain microbes are more tolerant to acidic conditions than others (Rousk et al., 2010). Soil water retention regulates moisture levels that are essential for microbial metabolism and activity (Schimel and Schaeffer, 2012). These physico-chemical factors directly influence soil fertility by affecting the availability of nutrients critical for microbial processes and plant-microbe interactions (Delgado-Baquerizo et al., 2016). In this study, we focused on total organic carbon and available phosphorus as indicators of soil fertility and microbial substrate availability, which are essential for microbial metabolism (Kögel-Knabner, 2002). Additionally, sodium, potassium, and calcium were examined for their roles in osmotic regulation, enzymatic activity, and soil structure stabilization, respectively (Marschner, 2012), while nitrate was assessed as a vital nitrogen source driving microbial growth and nutrient cycling (Hart et al., 1994). Understanding how these parameters interact can help clarify their effects on soil health and microbial function, which are critical for sustaining fertility in challenging environments.

In this study, we hypothesize that *T. gallica* L. promotes microbial diversity and enhances soil fertility in harsh environments such as salt flats. To evaluate it, we focused on the following specific objectives: (i) assess the impact of soil physico-chemical properties and nutrient content on microbial community structure, with a focus on their role in shaping microbial diversity, (ii) investigate bacterial community composition to identify dominant taxa and their ecological roles in nutrient cycling and soil adaptation; and finally, (iii) analyze relations between bacterial taxa and soil properties, to uncover key interactions informing restoration strategies.

## Materials and methods

### Site description and sample collection

**Figure 1:**
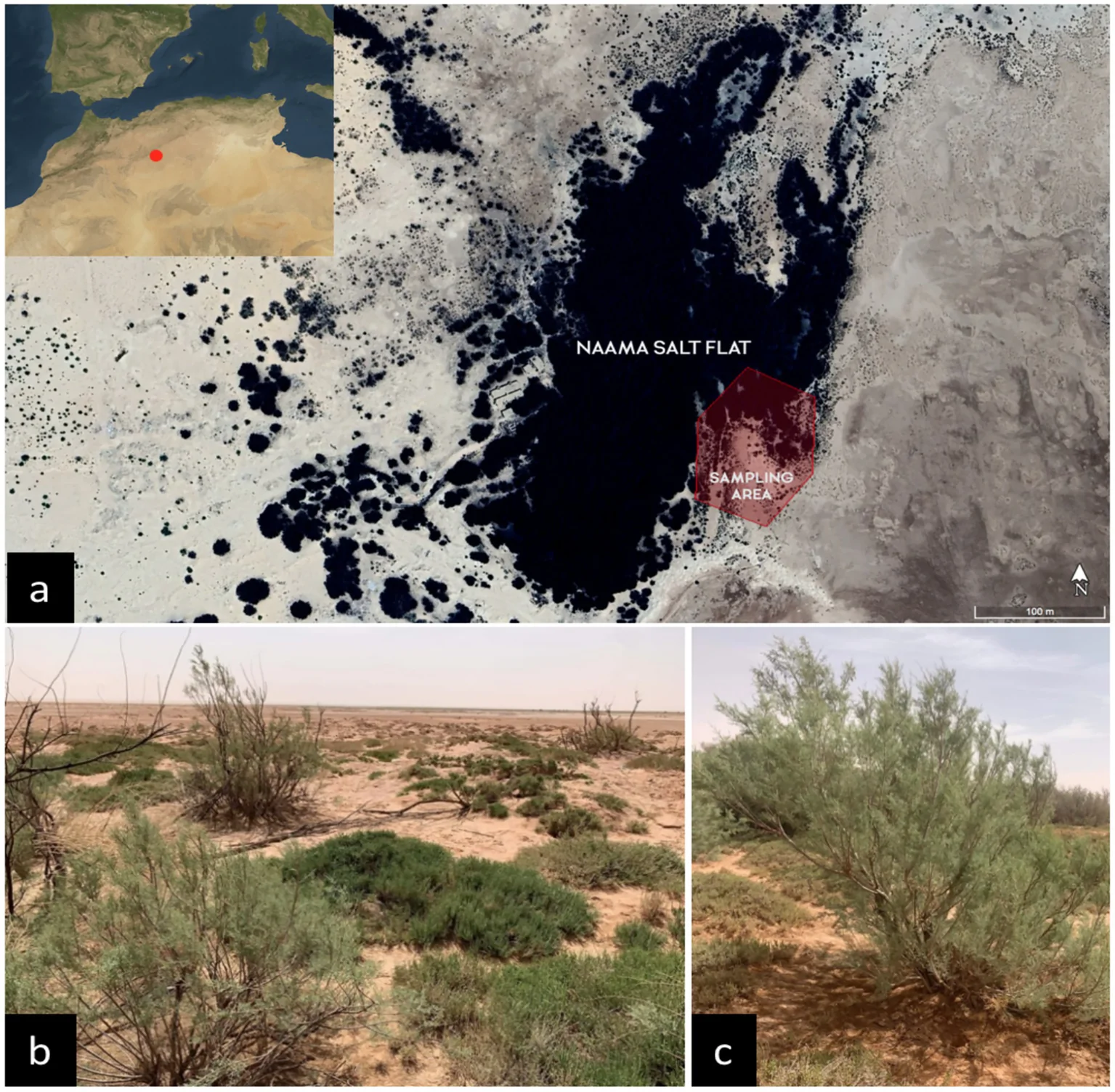
**(a)** Salt flat of Naama: mapped sampling area overview. **(b)** Landscape overview of Namaa’s salt flat. **(c)** *Tamarix gallica* L. in the salt flat of Namaa. Source: Google Earth. License: Used in accordance with the Google Earth/Google Maps Terms of Service.

The field experiment was conducted on 18 June 2023, at the salt flat of Naama, being part of the high plains of southern Oran, Algeria, extending from 32°08′ to 34°22′ northern latitude and from 0°36′ east to 0°46′ west longitude (Figure 1A). The salt flat of Naama is characterized by a relatively homogeneous landscape with altitudes ranging from 1,140 to 1,273 m.a.s.l., with a slope that ranges from completely flat to 7% (Bensaid and Boucherit, 2006).

The climate of the salt flat of Naama is arid to semi-arid, with increasing aridity from north to south, characterized by a dry summer season (Guerine et al., 2019). The annual rainfall varies between 124 and 352 mm, averaging 214.3 mm, and occurs principally in October with an average precipitation of 40.6 mm based on data from the Naama meteorological station, covering the period from 1996 to 2012. The mean annual temperature is approximately 15 °C, with recorded extremes ranging from a high of 41 °C in July 2012 to a low of −7 °C in February 2003 (Derdour et al., 2020).

As is typical of salt flat environments, the one of Naama is primarily saline and alkaline, characterized by poor drainage and high salt content due to saline groundwater evaporation (Derdour et al., 2020). A shallow aquifer occasionally feeds temporary saline ponds, but rainwater infiltration is limited by low soil permeability, leading to rapid evaporation and salt deposition (Benaradj et al., 2020). In the study area, the native vegetation includes *Tamarix* species (Figure 1B), which are halophytic plants commonly found in saline and arid environments (Chenchouni et al., 2015). This study focused on *Tamarix gallica* L. (Figure 1C), known for its salinity tolerance, soil stabilization, and contribution to ecosystem resilience in salt flats (Sghaier, 2023).

Soil samples from the rhizosphere of *T. gallica* plants (*n* = 3) and nearby bulk soil (*n* = 3) were collected. The samples were taken from 0 to 10 cm depth using a soil auger (Ø 7 cm) and collected in 50 mL sterile tubes. Samples were stored at 4 °C and sent in a cooler box to the laboratory where they were homogenized by sieving (∅ < 2 mm) for subsequent physicochemical and microbiological analysis.

### Soil physico-chemical properties and nutrient content

Soil water retention capacity (pF at 1500 and 33 kPa) and available water (AW) content were assessed using the Richard’s membrane method (Richards, 1941). Total Organic Carbon (TOC) was quantified using a colorimetric method described by (Mingorance et al., 2007) using a Spectronic Helios Gamma UV–Vis spectrophotometer (Thermo Fisher Scientific, Waltham, MA, USA). Available phosphorus (AP) was measured following the Olsen and Watanabe method (Olsen and Watanabe, 1957). The remaining analysis were measured from a 1:5 of soil:water (w/v) suspension: (i) soil pH using LAQUA PH1100 meter (HORIBA, Tokyo, Japan); (ii) electrical conductivity (EC) LAQUA EC1100 meter (HORIBA, Tokyo, Japan); (iii) sodium (Na^+^), potassium (K^+^), calcium (Ca^2+^); and nitrate (NO_3_^_^) ion contents using Horiba LAQUAtwin ionometers (HORIBA, Kyoto, Japan).

### Bacterial community composition, sequencing data processing and bioinformatics

Microbial DNA was extracted from 0.3 g of soil using DNeasy PowerSoil Kit (QIAGEN, Hilden, Germany) and quantified with a spectrophotometer ND-2000 Nanodrop (Thermo Fisher Scientific, USA). The V4–V5 regions (400–500 pb) of the bacterial 16S ribosomal RNA gene were amplified *in vitro* by polymerase chain reaction (PCR) using the 515FB/926R primer pair (Walters et al., 2016) and paired-end sequenced on an Illumina MiSeq platform, as described in (Comeau et al., 2017). One blank control was included to check for no contamination during DNA extraction. In turn, negative controls (1 per 96-well plate = 4 per MiSeq run) were analyzed and sequenced on the MiSeq to verify no substantial reads on this barcode combination.

Raw paired-end reads were processed with Quantitative Insights Into Microbial Ecology 2 (QIIME2 version 2019.7) software following the protocol established in Comeau et al. (2017). Reads were initially trimmed, joined, and quality filtered prior to denoising. Amplicon sequence variants (ASVs) were generated using the Deblur plugin (v. 1.1.0) implemented in QIIME2 tool, with sequences truncated to 360 bp before denoising. Deblur processing included dereplication, artifact removal, and chimera filtering.

After sequence processing, between 3.243 and 7.846 high-quality reads per sample were retained for downstream analyses, resulting in a total of 34.933 retained reads across all samples. Blank controls showed negligible read counts and were excluded from subsequent analyses. The number of final ASVs per sample ranged from 421 to 605. Representative ASV sequences were taxonomically assigned using the QIIME2 feature-classifier plugin with a naïve Bayes classifier trained against the SILVA database (release 132). Detailed sequencing statistics for each sample are provided in Supplementary Table S1.

Alpha- and beta-diversity analyses were conducted in QIIME2 using the final ASV table. For downstream statistical analyses, only bacterial taxa with relative abundance greater than 0.1% across all samples were retained.

### Statistical analysis

All statistical analyses were performed using PRIMER 7 (Clarke and Gorley, 2015) and R Studio (v. 4.1.3, R Core Team, 2020) softwares, with a significance level set at *p* ≤ 0.05. Data normality and homoscedasticity were assessed in R using the Shapiro–Wilk normality test and using Levene’s test, respectively. The Student’s *t*-test (or Mann–Whitney *U* test when normality was not met) was also performed in R to determine the effect of soil environment (bulk and rhizosphere soil) on physico-chemical properties, nutrient content and bacterial diversity indices.

To explore multivariate relationships among soil samples on physico-chemical and nutrient data, a Principal Coordinates Analysis (PCoA) was carried out in PRIMER 7 using Euclidean distance. This method allows for visualizing patterns in soil characteristics, identifying potential grouping of samples, and evaluating the influence of environmental variables on soil heterogeneity.

Differences in bacterial richness were evaluated using permutational multivariate analysis of variance (PERMANOVA; Anderson, 2001), based on Monte Carlo test.

To explore the influence of physico-chemical properties and nutrient content on bacterial taxa abundance in each soil environment, distance-based redundancy analysis (dbRDA) was performed in PRIMER 7. Since microbial communities in bulk soil are mainly shaped by soil properties and nutrients, while those in the rhizosphere are also influenced by plant roots, both factors were considered in the analysis. Similarity Percentage analysis (SIMPER) was used to determine the contribution of specific bacterial taxa to similarities and dissimilarities between rhizosphere and bulk soils, focusing on taxa with the highest contributions based on the Bray–Curtis similarity matrix (Clarke, 1993). Taxa contributing more than 1% to observed differences were selected for further analysis. Pearson correlation analyses (*p* < 0.1) were conducted in R Studio to examine the relationships between SIMPER-identified bacterial taxa and soil physico-chemical and nutrient content parameters. After removing redundant taxa, 52 bacterial taxa were used to generate the correlation visualizations using the *corrplot* and *RColorBrewer* R packages, with significant relationships focused on the genus levels. This integrative approach provided a comprehensive understanding of how environmental variables influence bacterial community composition and distribution (Friendly, 2002).

## Results

### Soil physico-chemical properties and nutrient content

Only available phosphorus (AP) was significantly higher in bulk soil (BS; *p* < 0.05) compared to rhizospheric soil (RS), (Table 1). Electrical conductivity (EC) and K^+^ content were higher in the rhizosphere, but the differences were not significant. Similarly, Na^+^ and NO_3_^−^ concentrations were higher in RS, but without significant differences (*p* > 0.05). Soil pH and TOC showed no differences between the two soil types. Water-related properties (pF at 1500 and 33 kPa) were also not significantly different, with both soils having similar available water percentages.

**Table 1:** Physico-chemical properties and nutrient content of bulk and rhizosphere soils.

Parameter	**BS**	**RS**
pH	8.47 ± 0.12 a	8.49 ± 0.37 a
EC (mS/cm)	1.30 ± 0.07 a	2.12 ± 0.92 a
pF (1,500 KPa)	3.33 ± 0.12 a	3.74 ± 0.18 a
pF (33 KPa)	23.7 ± 2.06 a	20.3 ± 0.39 a
AW (%)	20.36 ± 2.08 a	16.5 ± 0.30 a
TOC (%)	0.25 ± 0.10 a	0.25 ± 0.12 a
AP (mg/kg dry soil)	113.88 ± 21.41 a	12.36 ± 1.52 b
Na^+^ (ppm)	122.00 ± 13.31 a	214.66 ± 113.14 a
K^+^ (ppm)	18.66 ± 4.33 a	32.00 ± 6.42 a
Ca^2+^ (ppm)	38.66 ± 21.18 a	173.3 ± 49.10 a
NO_3_^−^ (ppm)	78.00 ± 8.72 a	163.30 ± 37.56 a

BS: bulk soil; RS: rhizosphere soil; EC: electrical conductivity; pF (1,500 kPa): soil water retention at permanent wilting point; pF (33 kPa): soil water retention at field capacity; AW: available water; TOC: total organic carbon; AP: available phosphorus; Na^+^: sodium; K^+^: potassium; Ca^2+^: calcium; NO_3_^−^: nitrate. Values show mean ± standard error (*n* = 3). Differences in lowercase letters denote statistically significant differences (*p* < 0.05).

Despite most of the individual parameters did not show statistical differences individually, the PCoA multivariate analyses clearly separated bulk and rhizosphere soils as shown in Supplementary Table S2. The first two axes explained 79.1% of the total variation (PCO1: 53.9%, PCO2: 25.2%). Rhizospheric soil (RS) samples are mainly positioned in upper and left quadrant and the bulk soil (BS) in the lower right quadrant.

**Figure 2:**
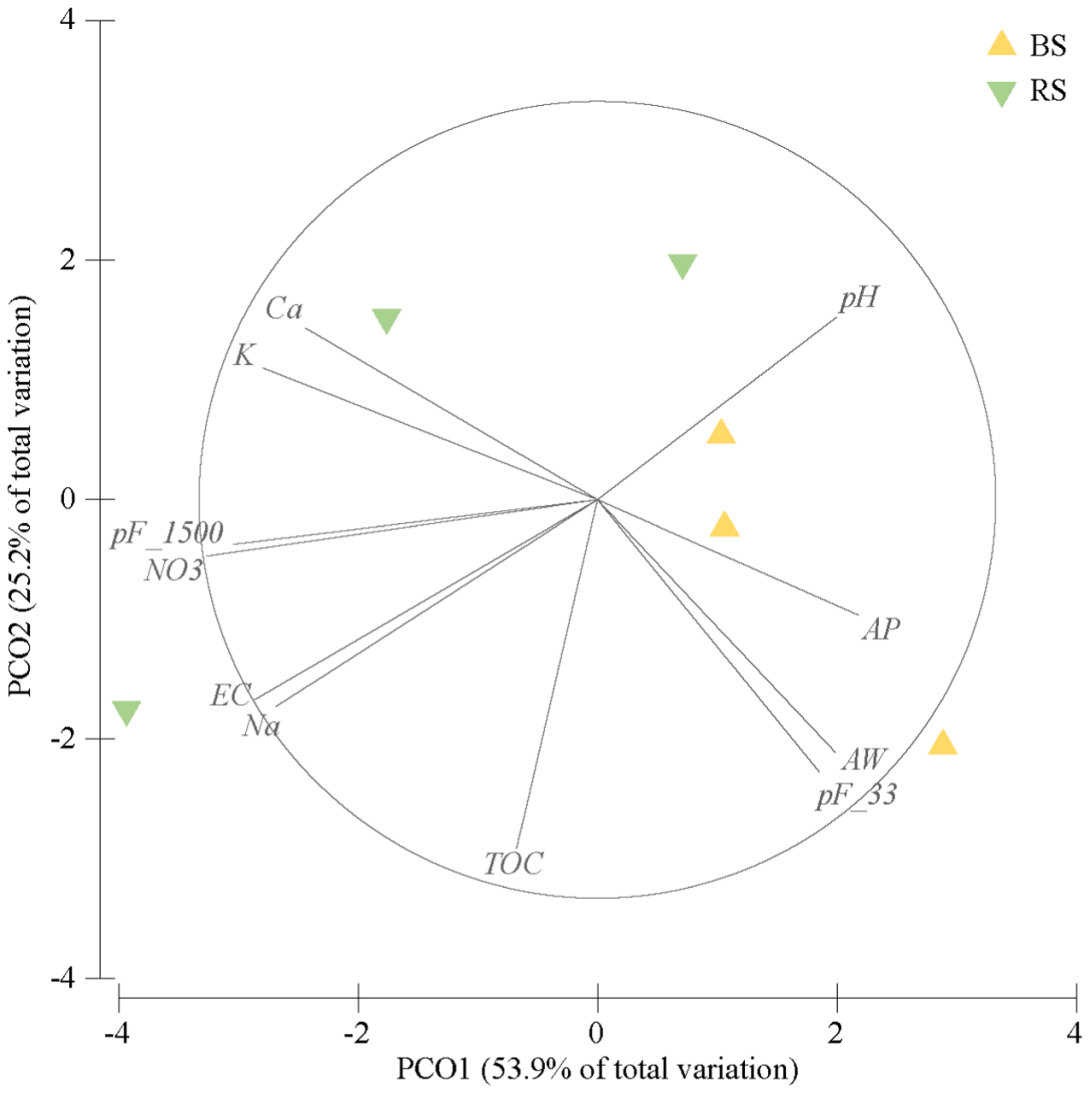
Principal coordinates analysis of physico-chemical properties in bulk and rhizosphere soils.

Besides, salinity, pF at 1500 KPa and the ion nutrients, K^+^, Ca^2+^, Na^+^ and NO_3_^−^ were strongly associated with rhizosphere soils, while AP, AW and pF at 33 KPa were strongly correlated with bulk soils. Finally, TOC and pH showed weak associations with both soils studied (Supplementary Table S2, Figure 2).

### Microbiological communities

#### Bacterial diversity

Alpha diversity indices showed higher values in BS vs. RS with statistical differences (*p* < 0.05) in all of them, apart from Faith (Table 2). Bulk soil showed more richness taxonomically (number of ASVs). The Shannon index accounts for both species richness and evenness, using a natural logarithm to quantify diversity. Pielou’s evenness measures how evenly individuals are distributed among species and Faith’s phylogenetic diversity, which considers the sum of branch lengths in a phylogenetic tree, was comparable between the two soil types.

**Table 2:** Alpha diversity indices of bacterial communities in bulk and rhizosphere soils.

Alpha diversity index	BS	RS
Richness (ASVs)	575 ± 15 a	461 ± 23 b
Faith	26.88 ± 0.89 a	22.17 ± 1.97 a
Shannon	8.44 ± 0.06 a	7.99 ± 0.02 b
Pielou	0.92 ± 0.00 a	0.90 ± 0.00 b

BS: bulk soil; RS: soil rhizosphere; ASVs: Amplicon Sequence Variants. Mean ± standard error (SEM). The lowercase letters denoting statistically significant differences (*p* < 0.05) were determined by *t*-test analysis.

#### Bacterial community composition

**Figure 3:**
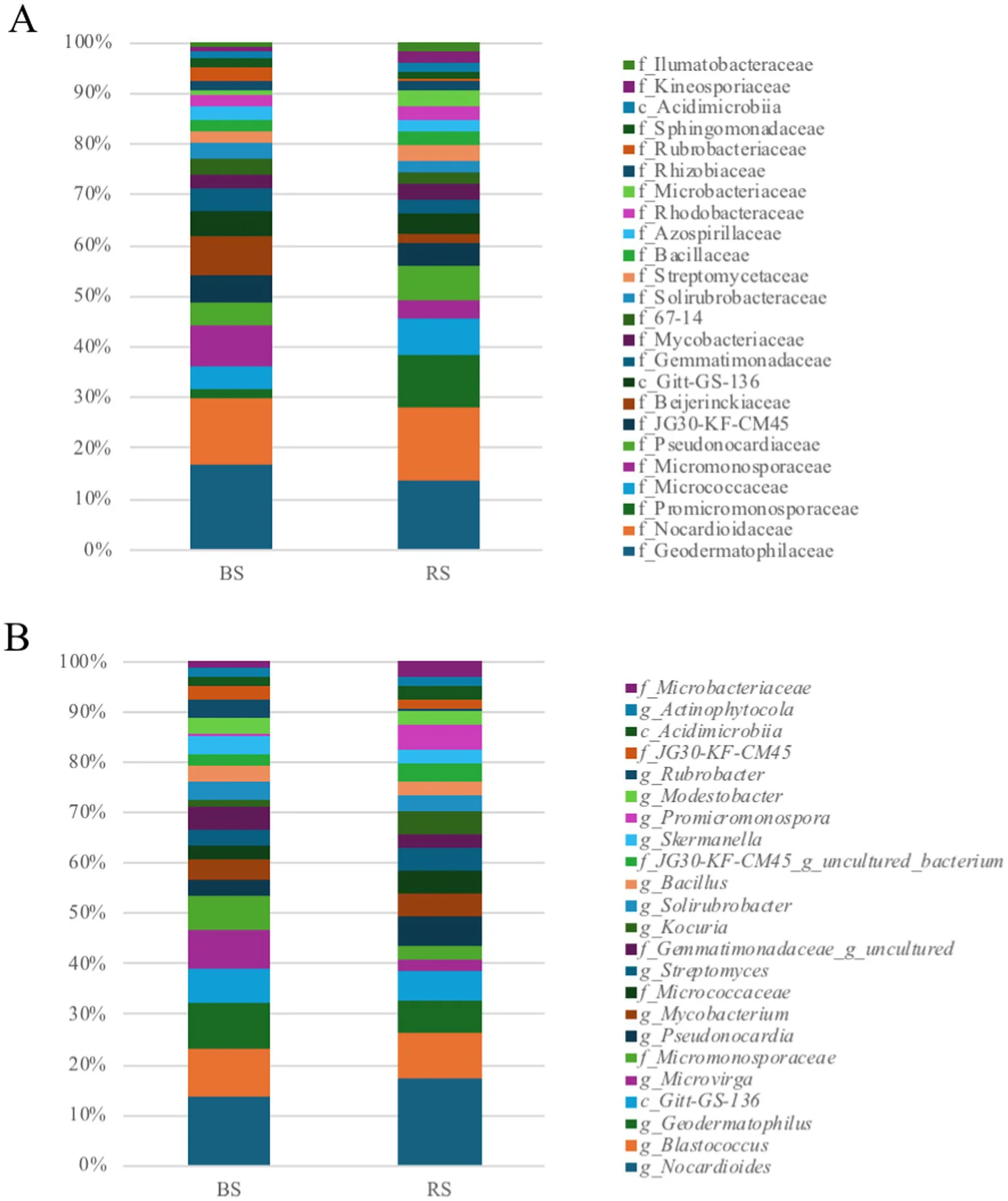
Bar chart analysis representing the most abundant bacterial taxa at family level (A; 24 bacterial taxa) and genus level or the next upper taxonomic level identified (B; 23 bacterial taxa) with relative abundance >1% distributed in bulk (BS) and rhizosphere soils (RS).

A total number of 90 bacterial taxa up to family or the next higher taxonomic level available were identified. The most abundant were *Geodermatophilaceae, Nocardioidaceae, Promicromonosporaceae, Micrococcaceae, and Micromonosporaceae,* although in different proportions in each environment (Figure 3). *Micromonosporaceae* was relatively more abundant in bulk soil, while *Promicromonosporaceae* was dominant in *T. gallica* rhizosphere soil.

At the genus or the next higher taxonomic level available, 148 taxa were identified (Figure 3). The most abundant taxa in both soil environments were *Nocardioides*, *Blastococcus*, *Geodermatophilus*, Gitt-GS-136 class, and *Microvirga*, although in different proportions in each environment. *Blastococcus*, *Geodermatophilus*, Gitt-GS-136 class *and Microvirga* were more relatively abundant in bulk soil while *Nocardioides* was dominant in rhizosphere soil.

**Figure 4:**
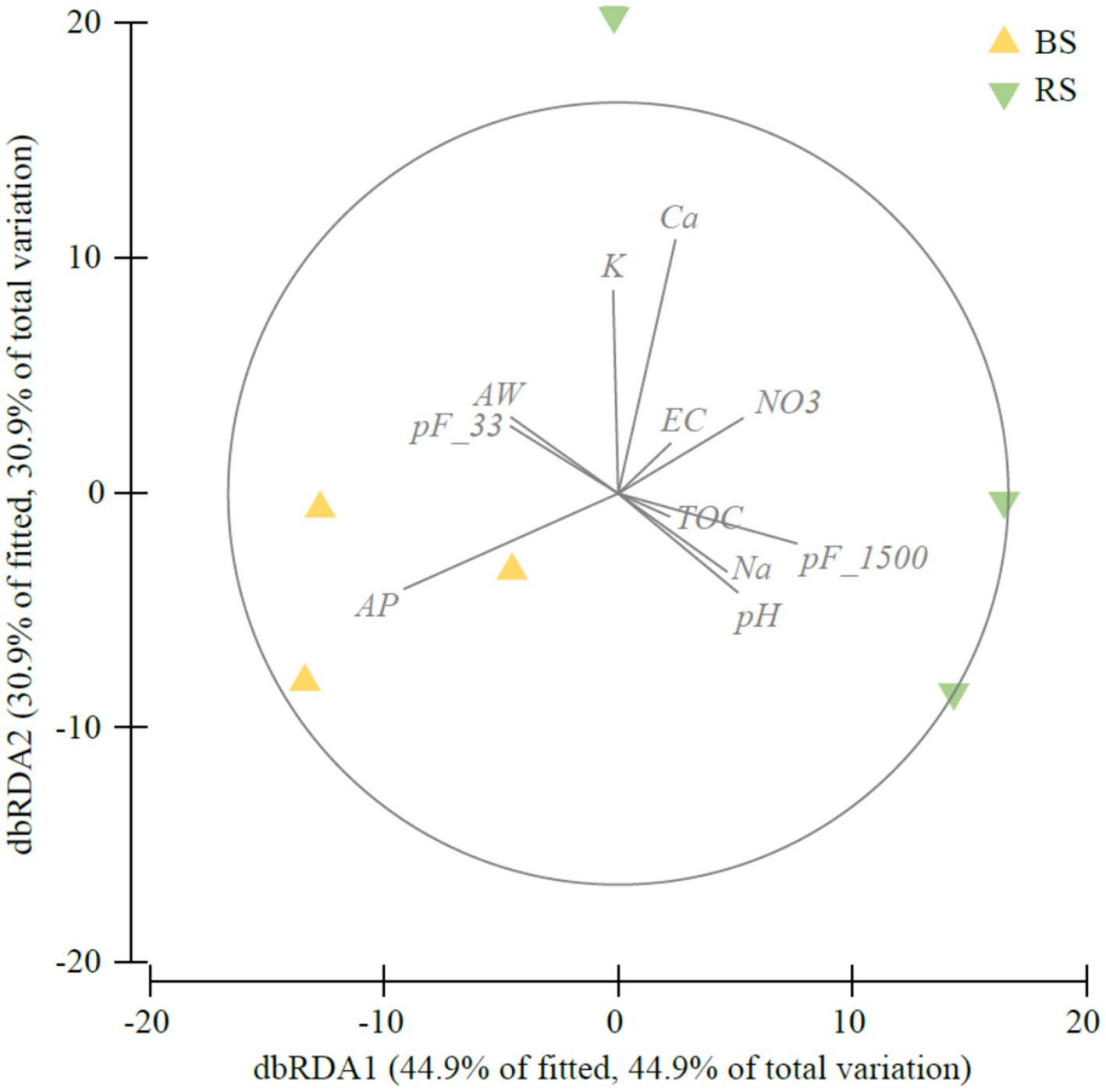
dbRDA analysis of genus-level composition in relation to physico-chemical properties in bulk and rhizosphere soils. BS: Bulk Soil; RS: Soil Rhizosphere; EC: electrical conductivity; pF (1,500 kPa): soil water retention at permanent wilting point; pF (33 kPa): soil water retention at field capacity; AW: available water; TOC: total organic carbon; AP: available phosphorus; Na^+^: sodium; K^+^: potassium; Ca^2+^: calcium; NO_3_^−^: nitrate. Arrows represent physico-chemical variables, with their length and orientation indicating the strength and direction of their correlation with sample separation.

The redundance analysis results based on the relative abundance of genera (or next taxonomically level classified) greater than 0.1% in all samples is shown in Figure 4. Two axes accounted for a cumulative 75.8% of the total variation (first axe explained 44.9% and second axe the 30.9% of the fitted variation), with physico-chemical variables represented indicating the strength and direction of their association with sample separation (Supplementary Table S3). Bulk and rhizosphere soil samples mainly separate along the dbRDA1 axis. Bulk soil microbial communities are negatively aligned with dbRDA1 and dbRDA2, and positively associated with AP. Rhizosphere communities were positively aligned with the dbRDA1 axis and strongly correlated with K^+^, Ca^2+^, pF at 1500 KPa, pH, Na^+^, and to a lesser extent NO_3_^−^, TOC, and EC. Finally, AW and pF at 33 KPa appear to exert a weaker influence on microbial composition of both soils.

#### Influence of *T. gallica* and soil properties on bacterial communities

SIMPER analysis revealed a high within-group similarity of 83.85% in bulk soils and 70.85% in rhizosphere soils, with 30 and 31 bacterial taxa, respectively, contributing more than 1%. In bulk soils, dominant genera taxa included *Nocardioides*, *Blastococcus*, and *Geodermatophilus*, while in the rhizosphere soils, *Nocardioides*, *Blastococcus*, and *Pseudonocardia* were the most dominant taxa.

The average dissimilarity between bulk and rhizosphere soils was 29.25%, driven by 19 bacterial taxa contributing more than 1%. Key contributors included *Promicromonospora*, *Microvirga*, *Rhodobacteraceae*, *Rubrobacter*, *Cellulosimicrobium*, and *Alphaproteobacteria*, with *Promicromonospora*, *Rhodobacteraceae*, *Cellulosimicrobium* and *Alphaproteobacteria* clustered in the rhizosphere, while *Microvirga* and *Rubrobacter* were more abundant in bulk soils. Detailed SIMPER contributions for within-group similarity and between-group dissimilarity are provided in Supplementary Table S4.

Overall, strong positive correlations were observed for taxa primarily found in the rhizosphere, including *Cellulosimicrobium* and *Isoptericola* with Na^+^ and EC; *Ilumatobacter* with Ca^2+^, K^+^, and NO_3_^−^; and *Kineococcus* with Ca^2+^ and K^+^. Conversely, *Ilumatobacter* exhibited a negative correlation with AP. In contrast, taxa predominantly associated with bulk soil, such as *Rubrobacter, Microvirga and Labrys* were strongly positively correlated with AP. Several strong correlations were observed among bacterial taxa contributing to rhizosphere and bulk soil dissimilarity. *Microvirga*, *Rubrobacter*, and *Labrys*, as well as Alphaproteobacteria with Rhodobacteriaceae exhibited strong positive associations.

## Discussion

The aim of this study is to investigate the bacterial composition and soil nutrient profiles of the bulk and rhizosphere soil associated with *Tamarix gallica* L., as well as their interactions in the largely unexplored salt flat of Naama, Algeria. Understanding how root presence may influence both physico-chemical properties and microbial communities is important for elucidating belowground ecological processes in halophilic systems.

Among the measured soil parameters, only available phosphorus (AP) differed significantly between bulk and rhizosphere soils, with higher values observed in bulk soil. This pattern may reflect phosphorus depletion in the rhizosphere due to plant uptake, potentially combined with microbial competition for available P. Similar rhizosphere-associated decreases in available phosphorus have been reported in other plant–soil systems (Richardson and Simpson, 2011). In saline and alkaline soils, phosphorus bioavailability may also be reduced through precipitation with calcium, thereby limiting soluble phosphorus pools (Shen et al., 2011). EC values indicated a slightly saline profile in both soil compartments (~1.3 to 2.12 mS/cm in Table 1), consistent with the temporal variability in salt distribution commonly observed in arid saline environments and the sandy-loam texture of the Sebkha of Naama (Rengasamy, 2006; Qadir et al., 2014). Similarly, Na^+^, K^+^, Ca^2+^, and NO_3_^−^ tended to be higher in rhizosphere soils, although these differences were not statistically significant. Notably, several of these variables exhibited substantial numerical differences between compartments. The absence of statistical significance may reflect the combined effects of within-group variability and limited replication, which can reduce the ability to detect differences between groups. Nevertheless, the observed trends remain consistent with the overall patterns revealed by the multivariate analyses and should be interpreted cautiously. The PCoA ordination showed a multivariate tendency for bulk and rhizosphere soils to occupy different regions of the ordination space, with salinity-related variables and ionic nutrients more associated with rhizosphere samples, and AP and water-related parameters more associated with bulk soil. The tendency towards higher EC and Na^+^ values in rhizosphere soils is consistent with the known salt-regulating mechanisms of halophytic species such as *T. gallica* (Flowers and Colmer, 2008; Sghaier, 2023). However, these interpretations remain hypothetical in the absence of significant differences and direct physiological measurements. TOC levels were similarly low in both compartments, which is consistent with the oligotrophic nature of arid saline conditions (Rath and Rousk, 2015).

The reduction in bacterial richness and evenness in rhizosphere soils, reflected by lower ASV richness, Shannon, and Pielou indices (Table 2), may result from selective pressures associated with the rhizosphere environment, where root exudates can favor specific microbial groups (Behr et al., 2023; Xavier et al., 2024). In contrast, bulk soils may support broader ecological niches and more taxonomically diverse communities, as commonly reported in arid and semi-arid ecosystems (Legeay et al., 2024). Faith’s phylogenetic diversity did not differ significantly between soil compartments.

PCoA analysis revealed a multivariate tendency for rhizosphere and bulk soil samples to occupy different regions of the ordination space (Figure 2), with rhizosphere soils tending to associate with salinity-related variables and ionic nutrients, whereas bulk soils showed a closer associate with AP and water-related parameters. The higher AP content observed in bulk soil may reflect plant uptake and rhizosphere-driven phosphorus depletion near roots (Richardson and Simpson, 2011), consistent with it being the only parameter showing statistically significant differences between soil compartments. In contrast, TOC and pH showed weak associations with both soil types in the ordination space.

The dbRDA analysis suggested possible associations between rhizosphere microbial communities and Na^+^, K^+^, Ca^2+^, and pF at 1500 KPa (Figure 4). These associations may reflect the influence of ionic composition and water retention on microbial assemblages, as reported in other saline environments (Ma et al., 2023). Bulk soil communities were more closely associated with AP, whereas rhizosphere communities tended to correlate with ionic parameters, broadly consistent with the physicochemical trends observed between both soil compartments. In contrast, AW and pF at 33 KPa appeared to exert weaker influences on microbial compositions in both soils.

The observed community patterns were broadly consistent with the ordination-based differentiation of both soil types (Figures 2,4), with distinct bacterial groups tending to dominate each environment. Promicromonosporaceae was relatively more abundant in rhizosphere soils, consistent with previous reports describing members of this family as adapted to rhizosphere environments and saline conditions (Wei et al., 2024). In contrast, Geodermatophilaceae and Nocardioidaceae were more abundant in bulk soils, in agreement with their frequent occurrence in oligotrophic and arid environments (Astorga-Eló et al., 2020; Xie and Pathom-aree, 2021).

At the genus level, *Nocardioides* was enriched in rhizosphere soils, whereas *Geodermatophilus* and *Blastococcus* were more abundant in bulk soils. These taxa are commonly associated with stress tolerance and adaptation to arid environments (Greengrass, 2020; Mghazli et al., 2021).

**Figure 5:**
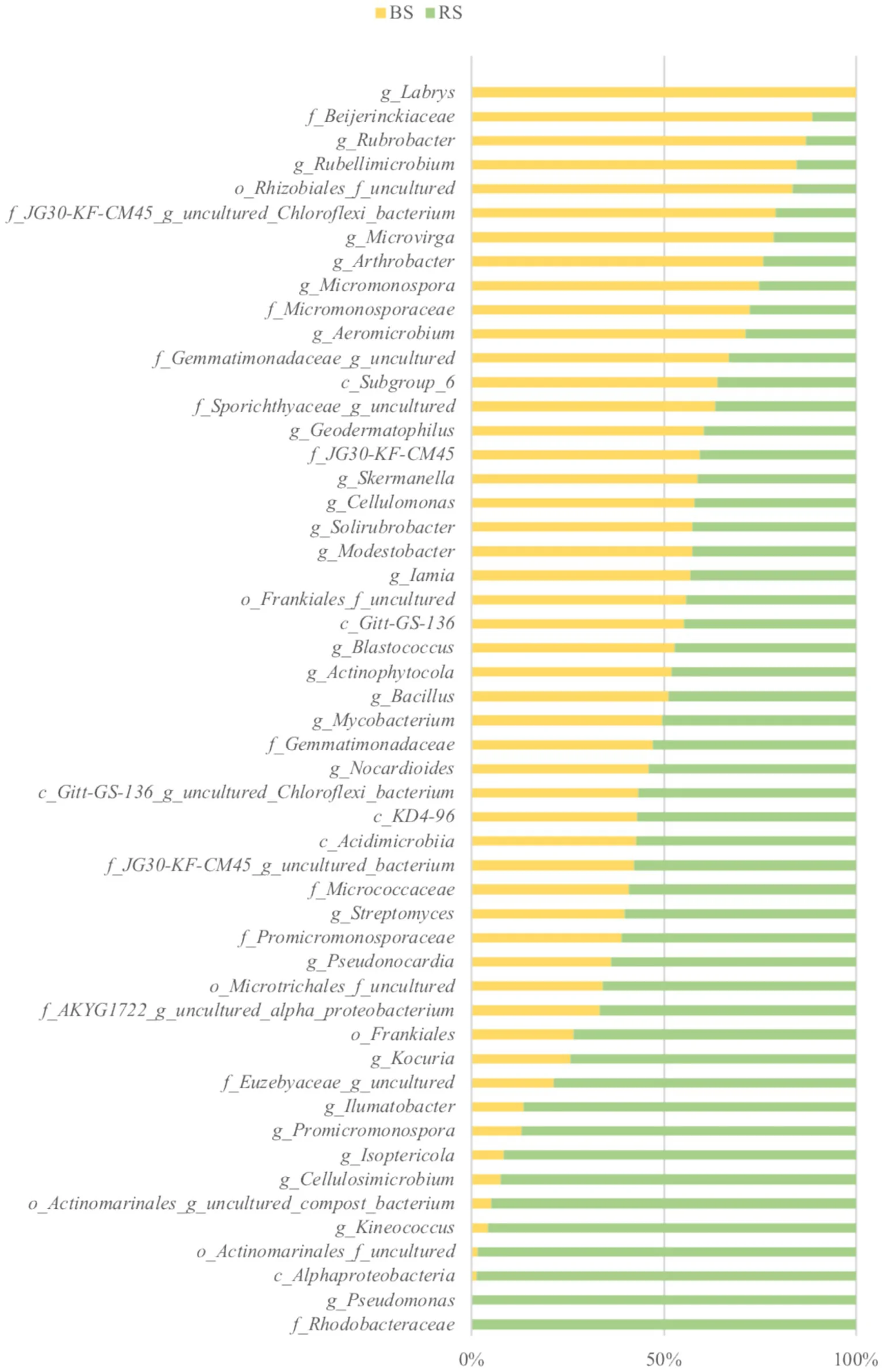
Comprehensive SIMPER analysis of most abundant bacterial genera across bulk (BS) and rhizosphere (RS) soils and their dissimilarity. The bacterial taxa shown were the ones that contributed more than 1% to similarity and dissimilarity. Letters before the name indicate the lowest taxonomic level of classification: o_: order, c_: class, f_: family, g_: genus. Bacterial taxa without letters in square brackets represent the genus level.

The SIMPER analysis revealed specific taxa contributing to community dissimilarity between bulk and rhizosphere soils (Figure 5). In rhizosphere soils, Rhodobacteraceae and *Promicromonospora* were relatively more abundant, which could be related to the capacity of certain members of these taxa to thrive under rhizosphere-associated and saline conditions (Drendel, 2024). Members of Rhodobacteraceae are frequently associated with metabolically versatile communities involved in organic matter turnover under dynamic environmental conditions (Gaj et al., 2025; Goszcz et al., 2025). Likewise, *Promicromonospora* has been described in saline and stress-prone environments in relation to polysaccharide degradation and osmotic stress tolerance (Yandigeri et al., 2012). In contrast, *Microvirga* and *Rubrobacter* were more abundant in bulk soils, consistent with their frequent occurrence in arid and nutrient-limited environments (Ferreira et al., 1999; Martínez-Hidalgo and Hirsch, 2017). *Rubrobacter* is particularly recognized for its tolerance to desiccation, radiation, and oxidative stress, traits commonly associated with extreme terrestrial habitats (Ferreira et al., 1999).

**Figure 6:**
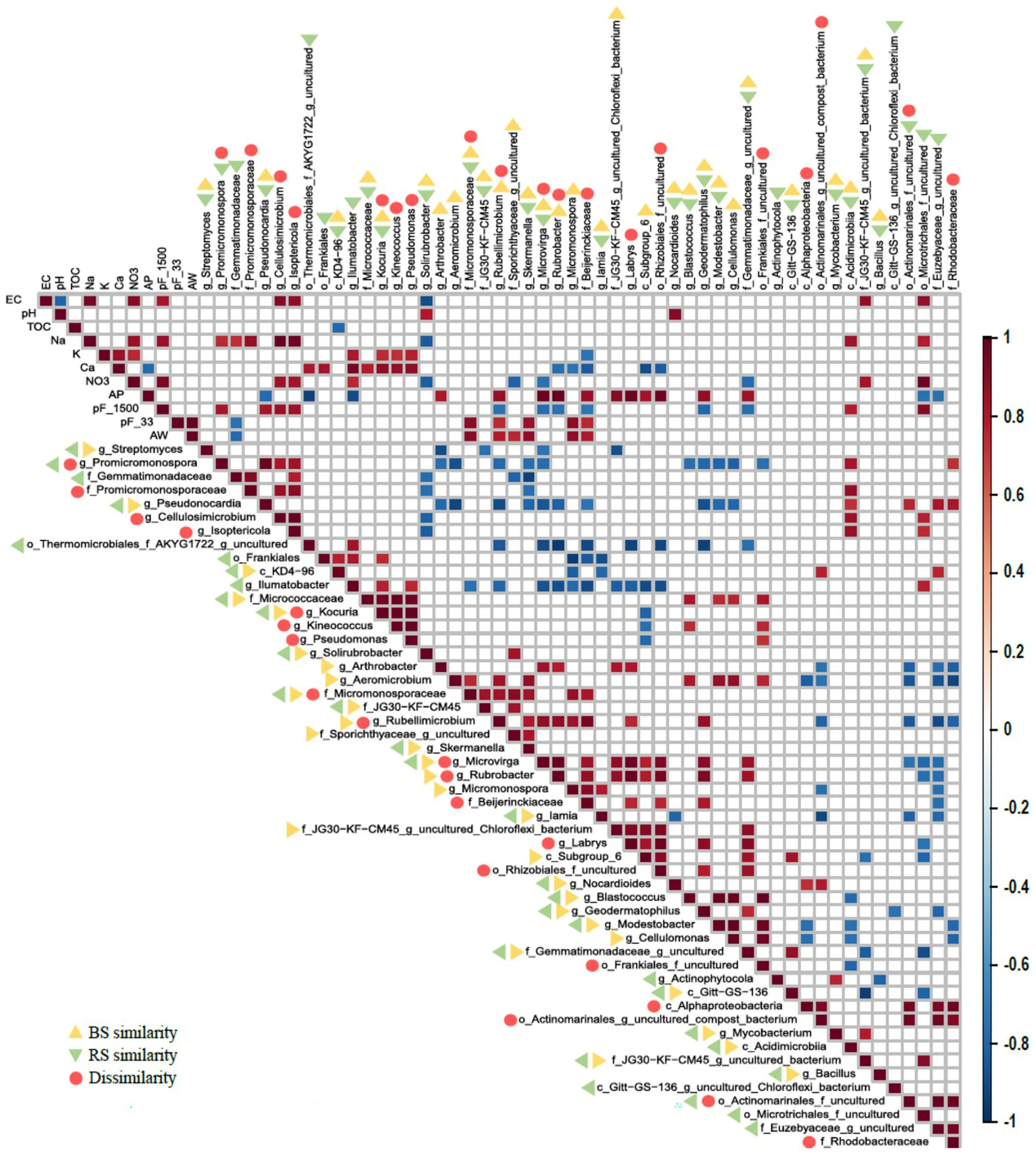
Unified genus-level Pearson correlation heatmap (*p* < 0.1): taxa contributing >1% from SIMPER in bulk soil (BS), rhizosphere soil (RS), and dissimilarity with soil properties. Footnotes: EC: electrical conductivity; pF (1,500 kPa): soil water retention at permanent wilting point; pF (33 kPa): soil water retention at field capacity; TOC: total organic carbon; AP: available phosphorus; Na^+^: sodium; K^+^: potassium; Ca^2+^: calcium; NO_3_^−^: nitrate. Red shades indicate positive correlations, while blue shades indicate negative correlations. The intensity of the color corresponds to the strength of the correlation, with darker shades representing stronger correlations.

Pearson correlations analyses revealed associations between taxa and physico-chemical soil properties in both soil compartments (Figure 6). In rhizosphere soils, *Cellulosimicrobium* and *Isoptericola* were positively correlated with Na^+^ and EC, consistent with previous reports describing halotolerant members of these genera in saline environments (Qingwei et al., 2023; Devi et al., 2024). *Cellulosimicrobium* has also been associated with phosphate solubilization and organic matter degradation under saline conditions (Nabti et al., 2014), whereas *Isoptericola* has been reported in relation to chitin degradation and plant-associated microbial activity (Alghamdi et al., 2023). *Ilumatobacter* showed a negative correlation with AP and positive correlations with Ca^2+^, K^+^, and NO_3_^−^, and has previously been linked to carbon cycling processes in soil environments (Chen et al., 2024). *Kineococcus* was positively associated with Ca^2+^ and K^+^, in agreement with reports describing stress-tolerant representatives of this genus in extreme terrestrial habitats (Williamson et al., 2024).

In bulk soils, *Rubrobacter*, *Microvirga*, and *Labrys were* positively correlated with AP. *Rubrobacter* is widely recognized for its tolerance to desiccation, radiation, and oxidative stress in arid soils (Ferreira et al., 1999). *Microvirga* has been reported in nutrient-limited and oligotrophic environments with active nitrogen cycling (Radl et al., 2014), and Labrys has been detected in arid biological soil crusts and low-input terrestrial systems (You et al., 2021).

Finally, positive associations were observed among several bacterial taxa, which may have contributed to the tendencies observed in the ordination-based separation between bulk and rhizosphere soils. *Microvirga*, *Rubrobacter*, and *Labrys* co-occurred predominantly in bulk soils, while *Alphaproteobacteria* and Rhodobacteraceae showed overlapping distribution patterns in rhizosphere-associated environments, consistent with previously reported co-occurrence patterns in similar systems (Ma et al., 2022; Zhao et al., 2024).

## Conclusion

This study highlights differences in microbial and physico-chemical characteristics between bulk and rhizosphere soils associated with *Tamarix gallica* L. in the saline environment of the Naama salt flat, Algeria. The rhizosphere showed trends toward higher ion concentrations (Na^+^, K^+^, and Ca^2+^) and lower bacterial diversity, whereas bulk soils retained higher available phosphorus (AP), the only parameter showing statistically significant differences between soil compartments. Microbial community patterns also differed between soil compartments, with rhizosphere soils showing higher relative abundance of taxa commonly associated with saline and plant-associated environments, including *Promicromonospora*, Rhodobacteraceae, and *Nocardioides*, while bulk soils were characterized by stress-tolerant taxa such as *Rubrobacter*, Geodermatophilaceae, and *Microvirga*.

Correlation analyses suggested associations between microbial composition and soil ionic properties in rhizosphere soils, whereas AP was more closely associated with bulk soil taxa. Positive co-occurrence patterns were also observed among several bacterial taxa within each soil compartment, possibly reflecting shared environmental preferences.

These findings should be interpreted in the context of the exploratory nature of this study and its limited sample size. Future research should focus on elucidating the molecular mechanisms underlying microbial adaptations in saline ecosystems and expanding sampling efforts to strengthen the robustness of these findings. Nevertheless, this work contributes rare microbial data from Algerian saline ecosystems, a region underexplored despite being severely impacted by soil salinization. The observed association between native halophytes and soil microbial communities may provide a basis for future research into plant growth-promoting rhizobacteria (PGPR) adapted to arid and saline conditions. Such findings could have potential relevance for researchers in microbial ecology, land rehabilitation programs, and sustainable agriculture in similar environments.

## Data Availability

The datasets presented in this study have been deposited in the NCBI Sequence Read Archive (SRA) under BioProject accession PRJNA1464001. The BioProject record is available at: https://www.ncbi.nlm.nih.gov/bioproject/PRJNA1464001.

## Glossary

16S rRNA: Ribosomal RNA 16S
A: Asterisk (for significance testing)
AP: Available Phosphorus
ASVs: Amplicon Sequence Variants
AW: Available Water
bp: Base Pairs (used in reference to DNA sequence length)
BS: Bulk Soil
Ca^2+^ Calcium
dbRDA: Distance-based Redundancy Analysis
EC: Electrical Conductivity
EPS: Exopolysaccharides
K^+^: Potassium
MA: Mean Annual
m.a.s.l.: Meters Above Sea Level
Na^+^: Sodium
NO_3_^−^ Nitrate
PCR: Polymerase Chain Reaction
PCoA: Principal Coordinates Analysis
PERMANOVA: Permutational Multivariate Analysis of Variance
PGPR: Plant Growth-Promoting Rhizobacteria
QIIME2: Quantitative Insights Into Microbial Ecology 2
R: Statistical software (R Studio)
RS: Rhizosphere Soil
SEM: Standard Error of the Mean
Shannon: Shannon-Wiener Index (biodiversity measure)
SIMPER: Similarity Percentage analysis
*T. gallica*: *Tamarix gallica*
TOC: Total Organic Carbon
UV: Ultraviolet
V4-V5: Regions of the 16S rRNA gene used for amplification
pF: Logarithm (base 10) of soil matric suction (hPa)
pH: Potential of Hydrogen (acidity/alkalinity of the soil)
t-test: Student’s *t*-test
