# Supplementary material for: Unveiling the bacterial community structure and soil properties in bulk and rhizospheric compartments of Tamarix gallica from an arid Algerian saline ecosystem
Source: Front Microbiol. 2026 Aug 10;17:1731684. doi: 10.3389/fmicb.2026.1731684 (PMC13501912; doi:10.3389/fmicb.2026.1731684)
Supplement: Supplementary file 1 [file Table_1.DOCX]

**Title**: Unveiling the Bacterial Community Structure and Soil Properties in Bulk and Rhizospheric Compartments of Tamarix gallica from an Arid Algerian Saline Ecosystem

**Authors**: Hanaa Abdelbari^1*,^ Natalia Rodríguez-Berbel^2 3 4^, Raúl Ortega^4^, Isabel Miralles^4^, Yassine Moustafa Mahdad^1 5^, Semir Bechir Suheil Gaouar^1^

**Affiliations**:

^1^ Applied Genetics in Agriculture, Ecology and Public Health Laboratory, University of Tlemcen, Tlemcen, Algeria

^2^ Department of Ecology and Ecosystem Management, TUM School of Life Sciences Weihenstephan, Chair of Soil Science, Technical University of Munich, Emil-Ramann-Straße 2, 85354 Freising, Germany

^3^ Research Unit Comparative Microbiome Analyses, Helmholtz Zentrum München, Ingolstädter Landstraße 1, 85764 Neuherberg, Germany

^4^ Department of Agronomy & Center for Intensive Mediterranean Agrosystems and Agri-Food Biotechnology (CIAIMBITAL), University of Almeria, E-04120 Almería, Spain

^5^ Laboratory for Sustainable Management of Natural Resources in Arid and Semi-Arid Areas, University Center of Naâma, Naâma, Algeria

* Corresponding author: [hanaa.abdelbari@univ-tlemcen.dz](mailto:hanaa.abdelbari@univ-tlemcen.dz)

# Supplementary material

**Table S1**. Sequencing quality statistics and read retention after Deblur processing.

| **Sample** | **Raw reads** | **Reads after Deblur** | **Chimeric reads removed** | **Final retained reads** | **Observed ASV** |
| --- | --- | --- | --- | --- | --- |
| **BS1** | 19325 | 7966 | 120 | 7846 | 560 |
| **BS2** | 19184 | 7257 | 116 | 7141 | 605 |
| **BS3** | 13444 | 4963 | 90 | 4873 | 559 |
| **RS1** | 15711 | 6056 | 109 | 5947 | 461 |
| **RS2** | 15756 | 6057 | 174 | 5883 | 502 |
| **RS3** | 8333 | 3290 | 47 | 3243 | 421 |
| **Blank** | 4 | 0 | 0 | 0 |  |

**Table S2**. Principal Coordinates Analysis

| *Percentage of variation explained by individual axes* | | | | |  |
| --- | --- | --- | --- | --- | --- |
| Axis | | Individual | Cumulative | |  |
| 1 | | 53.88 | 53.88 | |  |
| 2 | | 25.24 | 79.12 | |  |
| *Principal Coordinates* |  | | |  | |
| Sample | Axis 1 | | | Axis 2 | |
| BS1 | 10,349 | | | 0,53845 | |
| BS2 | 1,063 | | | -0,23138 | |
| BS3 | 28,872 | | | -20,562 | |
| RS1 | 0,71212 | | | 19,796 | |
| RS2 | -39,352 | | | -17,531 | |
| RS3 | -1,762 | | | 15,226 | |
| Footnotes: BS: Bulk Soil; RS: Soil Rhizosphere | | | | | |

**Table S3.** Distance-based redundancy analysis results, explanatory variance and contribution of relative abundance of soil bacterial taxa, physico-chemical properties, and nutrient content.

| *Percentage of variation explained by individual axes (% explained variation out of total variation)* | | | | |  |
| --- | --- | --- | --- | --- | --- |
| Axis | | Individual | Cumulative | |  |
| 1 | | 44.94 | 44.94 | |  |
| 2 | | 30.89 | 75.84 | |  |
| *Weights* |  | | |  | |
| *(Coefficients for linear combinations of X's in the formation of dbRDA coordinates)* | | | | | |
| Variable | dbRDA1 | | | dbRDA2 | |
| pH | 0.308 | | | -0.254 | |
| EC | 0.135 | | | 0.129 | |
| pF 1500 KPa | 0.459 | | | -0.128 | |
| pF 33 KPa | -0.277 | | | 0.173 | |
| AW | -0.276 | | | 0.196 | |
| TOC | 0.133 | | | -0.059 | |
| AP | -0.550 | | | -0.244 | |
| Na⁺ | 0.280 | | | -0.201 | |
| K⁺ | -0.013 | | | 0.520 | |
| Ca²⁺ | 0.147 | | | 0.650 | |
| NO₃⁻ | 0.321 | | | 0.193 | |
| Footnotes: pH: soil pH; EC: electrical conductivity; pF (1500 kPa): soil water retention at permanent wilting point; pF (33 kPa): soil water retention at field capacity; TOC: total organic carbon; AP: available phosphorus; Na⁺: sodium; K⁺: potassium; Ca²⁺: calcium; NO₃⁻: nitrate. | | | | | |

**Table S4**. SIMPER shows the relative abundance of the contribution of bacterial taxa with the lowest level of classification, the total and cumulative contribution to the average similarity as a percentage. Degree of similarity for each treatment. Contributions of bacterial taxa to mean percent similarity less than 1% are not shown.

| **Bacterial Taxa** | **Abundance (%)** | **Contribution (%)** | **Cumulative Contribution (%)** |
| --- | --- | --- | --- |
| BS average similarity: 83,85 % |  |  |  |
| *Nocardioides* | 2,69 | 3,22 | 3,22 |
| *Blastococcus* | 2,26 | 2,74 | 5,96 |
| *Geodermatophilus* | 2,22 | 2,63 | 8,60 |
| *Microvirga* | 2,04 | 2,42 | 11,02 |
| c_Gitt-GS-136 | 1,88 | 2,24 | 13,26 |
| f_Micromonosporaceae | 1,86 | 2,17 | 15,43 |
| f_Gemmatimonadaceae_g_uncultured | 1,55 | 1,75 | 17,19 |
| *Mycobacterium* | 1,46 | 1,68 | 18,87 |
| *Solirubrobacter* | 1,36 | 1,64 | 20,51 |
| *Rubrobacter* | 1,44 | 1,63 | 22,14 |
| *Skermanella* | 1,35 | 1,60 | 23,74 |
| f_Micrococcaceae | 1,27 | 1,60 | 25,34 |
| *Pseudonocardia* | 1,28 | 1,55 | 26,89 |
| *Modestobacter* | 1,24 | 1,47 | 28,36 |
| f_JG30-KF-CM45 | 1,16 | 1,41 | 29,78 |
| *Streptomyces* | 1,22 | 1,40 | 31,18 |
| *Rubellimicrobium* | 1,15 | 1,36 | 32,54 |
| f_JG30-KF-CM45_g_uncultured_bacterium | 1,14 | 1,31 | 33,85 |

| **Bacterial Taxa** | **Abundance (%)** | **Contribution (%)** | **Cumulative Contribution (%)** |
| --- | --- | --- | --- |
| RS average similarity: 70,85 % |  |  |  |
| *Nocardioides* | 2,91 | 4,15 | 4,15 |
| *Blastococcus* | 2,09 | 2,73 | 6,89 |
| *Geodermatophilus* | 1,80 | 2,68 | 9,57 |
| *Pseudonocardia* | 1,69 | 2,41 | 11,97 |
| c_Gitt-GS-136 | 1,68 | 2,35 | 14,33 |
| *Streptomyces* | 1,52 | 2,33 | 16,66 |
| *Mycobacterium* | 1,48 | 2,09 | 18,75 |
| *Bacillus* | 1,25 | 1,88 | 20,63 |
| *Kocuria* | 1,48 | 1,74 | 22,38 |
| f_JG30-KF-CM45_g_uncultured_bacterium | 1,31 | 1,72 | 24,10 |
| f_Micrococcaceae | 1,44 | 1,72 | 25,82 |
| f_Micromonosporaceae | 1,16 | 1,72 | 27,54 |
| *Skermanella* | 1,12 | 1,61 | 29,15 |
| *Promicromonospora* | 1,46 | 1,50 | 30,64 |
| *Microvirga* | 1,06 | 1,49 | 32,13 |
| f_JG30-KF-CM45 | 0,96 | 1,48 | 33,61 |
| *Solirubrobacter* | 1,14 | 1,48 | 35,09 |
| c_KD4-96 | 1,03 | 1,45 | 36,54 |
| f_Gemmatimonadaceae_g_uncultured | 1,08 | 1,39 | 37,93 |
| o_Actinomarinales_f_uncultured | 1,07 | 1,29 | 39,22 |
| o_Microtrichales_f_uncultured | 0,94 | 1,29 | 40,51 |
| f_Euzebyaceae_g_uncultured | 0,97 | 1,28 | 41,80 |
| *Ilumatobacter* | 0,85 | 1,23 | 43,03 |
| c_Gitt-GS-g_uncultured_Chloroflexi_bacterium | 0,90 | 1,23 | 44,26 |
| *Iamia* | 0,84 | 1,19 | 45,44 |
| f_AKYG1722_g_uncultured_alpha_proteobacterium | 0,76 | 1,18 | 46,62 |
| c_Acidimicrobiia | 1,04 | 1,12 | 47,74 |
| *Modestobacter* | 1,00 | 1,09 | 48,83 |
| o_Frankiales | 0,79 | 1,05 | 49,88 |
| f_Gemmatimonadaceae | 0,68 | 1,03 | 50,90 |
| *Actinophytocola* | 0,93 | 1,02 | 51,93 |

| **Bacterial Taxa** | **BS Abundance (%)** | **RS Abundance (%)** | **Contribution (%)** | **Cumulative Contribution (%)** |
| --- | --- | --- | --- | --- |
| BS & RS average dissimilarity: 29,25 % | | | | |
| *Promicromonospora* | 0,53 | 1,46 | 1,90 | 1,90 |
| o_Actinomarinales_f_uncultured | 0,08 | 1,07 | 1,85 | 3,75 |
| *Microvirga* | 2,04 | 1,06 | 1,84 | 5,59 |
| f_Rhodobacteraceae | 0,00 | 0,97 | 1,81 | 7,39 |
| c_Alphaproteobacteria | 0,08 | 0,93 | 1,69 | 9,08 |
| *Rubrobacter* | 1,44 | 0,56 | 1,65 | 10,72 |
| *Cellulosimicrobium* | 0,36 | 0,87 | 1,51 | 12,23 |
| *Isoptericola* | 0,29 | 0,90 | 1,49 | 13,73 |
| *Rubellimicrobium* | 1,15 | 0,39 | 1,41 | 15,14 |
| f_Micromonosporaceae | 1,86 | 1,16 | 1,32 | 16,46 |
| *Kineococcus* | 0,17 | 0,68 | 1,21 | 17,67 |
| o_Actinomarinales_g_uncultured_compost_bacterium | 0,10 | 0,73 | 1,19 | 18,86 |
| f_Promicromonosporaceae | 0,69 | 0,75 | 1,18 | 20,04 |
| *Kocuria* | 0,92 | 1,48 | 1,06 | 21,10 |
| *Labrys* | 0,56 | 0,00 | 1,06 | 22,15 |
| *Pseudomonas* | 0,00 | 0,55 | 1,04 | 23,19 |
| o_Frankiales_f_uncultured | 0,90 | 0,65 | 1,02 | 24,21 |
| f_Beijerinckiaceae | 0,82 | 0,29 | 1,01 | 25,22 |
| o_Rhizobiales_f_uncultured | 0,84 | 0,31 | 1,00 | 26,22 |

BS: Bulk Soil; RS: Soil Rhizosphere. Letters before the name indicate the lowest taxonomic level of classification: o_: order, c_: class, f_: family, g_: genus. Bacterial taxa without letters in square brackets represent the genus level.
